# Alcohol and lactation: Developmental deficits in a mouse model

**DOI:** 10.3389/fnins.2023.1147274

**Published:** 2023-03-13

**Authors:** Roberto F. Perez, Kathleen E. Conner, Michael A. Erickson, Mirembe Nabatanzi, Kelly J. Huffman

**Affiliations:** ^1^Department of Psychology, University of California, Riverside, Riverside, CA, United States; ^2^Interdepartmental Neuroscience Program, University of California, Riverside, Riverside, CA, United States

**Keywords:** alcohol, behavior, neocortex, lactation, anatomy, brain development, postnatal neocortical development, lactational ethanol exposure

## Abstract

It is well documented that prenatal ethanol exposure *via* maternal consumption of alcohol during pregnancy alters brain and behavioral development in offspring. Thus, the Centers for Disease Control (CDC) advises against maternal alcohol consumption during pregnancy. However, little emphasis has been placed on educating new parents about alcohol consumption while breastfeeding. This is partly due to a paucity of research on lactational ethanol exposure (LEE) effects in children; although, it has been shown that infants exposed to ethanol *via* breast milk frequently present with reduced body mass, low verbal IQ scores, and altered sleeping patterns. As approximately 36% of breastfeeding mothers in the US consume alcohol, continued research in this area is critical. Our study employed a novel murine LEE model, where offspring were exposed to ethanol *via* nursing from postnatal day (P) 6 through P20, a period correlated with infancy in humans. Compared to controls, LEE mice had reduced body weights and neocortical lengths at P20 and P30. Brain weights were also reduced in both ages in males, and at P20 for females, however, female brain weights recovered to control levels by P30. We investigated neocortical features and found that frontal cortex thickness was reduced in LEE males compared to controls. Analyses of dendritic spines in the prelimbic subdivision of medial prefrontal cortex revealed a trend of reduced densities in LEE mice. Results of behavioral tests suggest that LEE mice engage in higher risk-taking behavior, show abnormal stress regulation, and exhibit increased hyperactivity. In summary, our data describe potential adverse brain and behavioral developmental outcomes due to LEE. Thus, women should be advised to refrain from consuming alcohol during breastfeeding until additional research can better guide recommendations of safe maternal practices in early infancy.

## Introduction

Alcohol is known as a developmental teratogen in mammalian systems. However, research in this area has primarily focused on exposures during the prenatal period. Maternal consumption of alcohol during pregnancy can result in Fetal Alcohol Spectrum Disorders (FASD) in offspring and children with FASD may exhibit physical, cognitive, emotional, and behavioral phenotypes related to the exposure (May et al., 2009, 2014; Hoyme et al., 2016). Thus, Centers for Disease Control (CDC) have released a statement that no amount of alcohol is safe to consume during pregnancy (Centers for Disease Control and Prevention, 2022a). Generally, these recommendations are followed, as demonstrated by a reduction in alcohol consumption during pregnancy. However, consumption levels approach preconception levels shortly after birth in some populations (Little et al., 1990; Giglia and Binns, 2006). The prevalence of breastfeeding mothers consuming alcohol is high, ranging from 20% in Canada (Popova et al., 2013), 36% in the United States (May et al., 2016), and 60% in Australia (Tay et al., 2017). For a specific example, in Seattle, Washington, 80% of women consumed alcohol during the month before conception, 40% consumed alcohol during the last trimester of pregnancy, and 70% were drinking 3 months postpartum. Notably, this study also reported that 10% of breastfeeding mothers reported drinking more than once a day (>15 g alcohol) (Little et al., 1990).

Given the prevalence of maternal alcohol consumption during breastfeeding, it is important to understand how this can represent a teratogenic exposure for infants. Studies have shown that the levels of alcohol in the breast milk mirror the amount of alcohol in the blood (Lawton, 1985; Chien et al., 2005). These levels peak at 30–60 min after ethanol consumption and continue to be detected 2–3 h after consumption (Chien et al., 2005; Centers for Disease Control and Prevention, 2019). Although these levels are lower than the percentage in alcoholic beverages, they are non-zero values. In infants, exposure to breast milk containing alcohol may result in reduced body mass and verbal IQ scores (May et al., 2016). Congruently, exposure to alcohol *via* breast milk may result in a dose-dependent reduction of cognitive functions as seen when testing exposed children aged 6–7 years (Gibson and Porter, 2018) and dose-dependent reductions in children’s academic abilities up to grade 5 (Gibson and Porter, 2020). Additionally, deficits in abstract reasoning skills are observed at age 7 in lactational-exposed children (Oei, 2019). Changes in sociability can also occur as exposed infants scored below, or within the monitoring zone, on the scale of the personal-social interactions at 12 months of age (Tay et al., 2017). Despite these potential negative effects of alcohol compromised breast milk on offspring development, there is a disconnect between conclusions drawn from scientific literature and behaviors in many new mothers.

In humans, there is variability in maternal behavior in terms of infant feeding preferences. In the US from 2012 to 2019, around 80% of mothers breastfed their infants, with just over half of them breastfeeding exclusively [from the National Immunization Survey (Centers for Disease Control and Prevention, 2019)]. Additionally, there is variability among women in their ability to metabolize alcohol and to respond to stressors, which can moderate infant exposure. Indeed, higher tolerance and stress may result in the increase of the consumption of alcohol, for certain populations (Guinle and Sinha, 2020). Women who consume alcohol during pregnancy are also more likely to drink while breastfeeding (May et al., 2016), suggesting certain populations may be considered high-risk for breast milk contamination. Additionally, unplanned, and drastic lifestyle changes may influence alcohol consumption levels. For example, the COVID-19 pandemic and subsequent “stay-at-home” orders, rapidly emerged as a public and/or personal health concern for many. In response to this novel stressor, women in the United States showed an increase in their Alcohol Use Disorders Identification Test scores during the COVID-19 “stay-at-home” order (Boschuetz et al., 2020). These results translate to an increase in frequency and quantity of alcohol ingested in those who already used alcohol; congruently, factors such as having children at home and a history of substance abuse were positively associated with an increase in alcohol use during the pandemic (Boschuetz et al., 2020). Similar results were observed in Australia (Bramness et al., 2021), Norway (Rossow et al., 2021), and Belgium (Vanderbruggen et al., 2020), and thus, the pandemic and “stay-at home” orders may have unintentionally increased infant alcohol exposure *via* increased maternal consumption. These studies show an increase in alcohol consumption in certain child rearing populations, elucidating the deleterious effects of postnatal ethanol exposure *via* breast milk, and bolster the importance of alcohol abstinence during breastfeeding. However, published postnatal alcohol exposure paradigms (*via* breast milk) tend to be uncontrolled, unstandardized, and often limited to humans. Much of the existing data leave questions of dosing, timing, and how the developing nervous system is affected by lactational ethanol exposure (LEE). Data from animal models are not always consistent, most likely due to the variability in postnatal ethanol exposure methods, ranging from direct ethanol exposure to combined prenatal and postnatal exposure. In one study, researchers exposed rat pups to ethanol *via* intragastric intubation from postnatal (P) day 4 to 8 and reported increased male body weights but no increases in cerebral cortex weight (Light et al., 1989). Another direct exposure study reported a reduction of stem cell progenitor cells in the hippocampus and reduced adult neurogenesis after a singular subcutaneous injection of alcohol at P7 (Ieraci and Herrera, 2007). A study from Vilaró et al. (1987) exposed rat pups to alcohol *via* an alcohol-treated mother and reported a reduction in weight of rat pups at age P15 compared to controls; however, this study exposed rats to ethanol during gestation as well as postnatally. These studies provide much-needed evidence toward the damaging effects of postnatal ethanol exposure; however, they do not target a particular time window in mammalian brain development. Hence, many of their results are contradictory. To combat this, an analogous age range for exposure must be established between mice and humans. To begin, the brain growth spurt (BGS) is a time window where the mammalian brain undergoes rapid growth (Dobbing and Sands, 1979). In humans this period ranges from the third trimester of pregnancy to about the first 2 years of life, peaking at the birth (Dobbing and Sands, 1979). In murine models, this period ranges from the first week postnatal to the third week, peaking around P7 (Dobbing and Sands, 1979). A study has shown that exposure to alcohol during the BGS induces deficits such as a reduction in long-term cerebellar growth and altered rotarod performance in a rat model (Goodlett et al., 1991). However, this study used artificial-rearing procedures to directly expose pups to ethanol during the P4–P9 time window and was a binge model (Goodlett et al., 1991). Furthermore, ethanol exposure has been shown to cause alterations in synaptic pruning (Kyzar et al., 2016). In mice, synaptic pruning reaches its peak 2–3 weeks postnatal (Lewis, 2011), this is within the BGS, providing further evidence of sensitivity toward perturbations early in postnatal development. Clearly, additional research is needed to illuminate the specific details of risk including dose-dependencies and the interaction of developmental time and exposure. Here, we are specifically interested in how maternal drinking while breastfeeding impacts brain and behavioral development of offspring. The exposure period we targeted is within the BGS but begins on a postnatal day roughly equivalent to the day of human birth, to better mimic the time when breastfeeding would begin in humans.

In the current study, we targeted early LEE in our mouse model by estimating typical human birth in murine time. When making cross-species comparisons for developmental stage, the first postnatal week in mice relates to the third trimester in humans (Clancy et al., 2007). As our study did not aim to model human *prenatal* alcohol exposure, or FASD, we began our maternal dosing of ethanol at the end of the first week of murine life (evening of postnatal day 6). This way, offspring will have consumed alcohol *via* breast milk by P7. Estimates of human day of birth (full term) is between 245 and 265 days post conception with the mouse equivalent between 7 and 9 days postnatal (Clancy et al., 2007; Jukic et al., 2013). Specifically, we exposed CD-1 pups to breast milk contaminated with ethanol, *via* maternal consumption, at the end of the postnatal week until weaning. By mimicking human postpartum drinking behavior, our results revealed potential effects of LEE on offspring outcomes. We measured maternal blood ethanol content to assure exposure validity and blood osmolality to assess hydration. We analyzed several outcome measures in offspring to determine to what degree ethanol exposure *via* lactation altered key features of neuroanatomical development and whether these phenotypes were read out in behavior. As predicted, LEE resulted in abnormal brain and behavioral development.

## Materials and methods

### Animal care

All breeding and experimental studies were conducted in accordance with protocol guidelines approved by the Institutional Animal Care and Use Committee (IACUC) at the University of California, Riverside (UCR). CD-1 mice, initially purchased from Charles River Laboratories (Wilmington, MA, USA), were used for breeding. We chose to use the outbred CD-1 mouse strain in this lactational model because these mice show superior maternal care compared to inbred strains and because we had validated them as a model for prenatal ethanol exposure (PrEE) in our prior work (El Shawa et al., 2013). Mice were housed in animal facilities located at UCR that were kept at approximately 22°C on a 12-h light/dark cycle. Mouse chow and water (for controls), or mouse chow and a 25% ethanol solution in water, were provided *ad libitum* to the dams according to the dosing schedule.

### Breeding and lactational ethanol exposure paradigm

**FIGURE 1:**
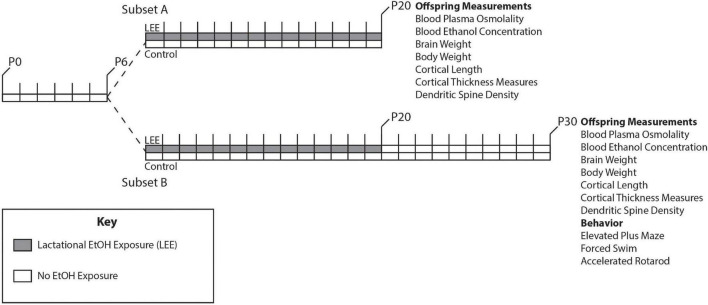
Experimental paradigm. Mice were designated as control or LEE at P6. LEE dams received 25% EtOH when pups were P6-P20. At P20 pups were weaned, divided into two subsets, and no longer exposed to EtOH. Subset A was subjected to a variety of measurements at P20. Subset B was subjected to measurements as well as behavioral tests at P30.

Adult female and male mice, aged P90-150, were paired just before the start of the dark cycle. Once a vaginal plug was detected, the male was removed from the cage. Throughout pregnancy, mouse chow and water were provided *ad libitum* to all dams. Dams were undisturbed through pregnancy and birth until the pups were 6 days old, when litter sizes were recorded (Figure 1). During this time, we pseudo-randomly assigned each dam to the control or experimental group (Lactational Ethanol Exposed, LEE group). LEE dams had their water replaced with a 25% v/v ethanol in water solution throughout the exposure period from the evening of P6 to P20, while control dams remained on water. The liquid bottle tip was placed high in the cage so that developing pups could not reach it, thus, their only liquid intake was *via* dam breast. There were no alterations to the dam’s food supply through the exposure period for any experimental condition. Measurements were taken daily for maternal liquid and food consumption during the exposure period for both conditions. At wean (P20), litter size was assessed, control and LEE pups were weighed and divided into two subsets. Subsets A and B had different sacrificial end dates of P20 and P30, respectively. Subset B control and LEE pups were weighed and subjected to no more than two behavioral assays. The division of the litters into subsets allowed us to evaluate the short and long-term effects of LEE with an array of techniques. To avoid litter effects, we distributed pups from multiple litters for each assay tested.

### Dam and pup blood ethanol concentration and plasma osmolality measurements

To measure dam and pup blood ethanol concentration (BEC) and blood plasma osmolality (pOsm), a measure of hydration, animals from control and LEE groups were subjected to a whole blood collection protocol. Whole blood was collected at the time of weaning for dams and pups *via* cardiac puncture. After collection, blood was placed in an untreated 1.5 ml centrifuge tube and allowed to clot for 30 min at room temperature. The entire sample was then centrifuged at 4,000 × *g* for 15 min at 4°C to separate serum from whole blood. To determine BEC in control and LEE groups, an alcohol dehydrogenase (ADH) based enzymatic assay (Pointe Scientific, Canton, MI, USA) was employed. In brief, ethanol, and nicotinamide adenine dinucleotide (NAD+) become catalyzed by ADH and this interaction causes the oxidation of ethanol to acetaldehyde and reduces NAD+ to NADH. The modified sample was read on a Nanodrop 2000 Spectrophotometer (Thermo Fisher Scientific) at 340 nm. To determine pOsm, freshly extracted serum from control and LEE groups were subjected to testing using an osmometer.

### Brain tissue preparation and collection

Pups from all conditions were randomly assigned for gross anatomical studies. Mice were weighed then sacrificed using a lethal dose of sodium pentobarbital (100 mg/kg) administered *via* intraperitoneal injection. Mice were transcardially perfused with 0.9% saline followed by 4% paraformaldehyde in PBS (PFA, pH: 7.4) for fixation. The skulls were post-fixed in a 4% PFA solution overnight, then the brains were extracted, weighed, and imaged. Dorsal views of whole brains were imaged using a Zeiss (Oberkochen, Germany) Axio high-resolution (HRm) camera attached to a dissecting microscope. Extracted brains were stored in 4% PFA for later use.

### Anatomical measurements

Brain and body weights were assessed at P20 and P30 for both sexes and conditions. They were compared using statistical analyses and a brain/body weight ratio was computed to determine if any changes in brain or body weight were independent of one another. Typically, in normal development, brain and body size/weight are related. Larger animals within the same species tend to have larger brains. We calculated the ratio able to differentiate whether the exposure was causing a decrease in brain size alone, or whether decreases in brain size from our perturbation could be related to overall decrease in body size. Next, to measure cortical length of all brains, we used a digital micrometer in ImageJ (NIH, Bethesda, MD, USA), using the dorsal whole-brain images. To examine anatomical cortical areas, perfused brain tissues were hemisected and cryoprotected using a 30% sucrose (w:v) in PBS solution. Tissue was then sectioned using a Leica cryostat at 40 μm thick in the coronal plane, mounted on subbed slides, and stained for Nissl bodies using a 0.1% Cresyl Violet solution staining protocol then imaged using a Zeiss Axio Upright Imager microscope equipped with a Zeiss Axio HRm camera. To control for comparisons between groups, the Allen Mouse Brain Atlas (Allen Institute for Brain Science, 2004)^1^ and the Paxinos Developing Mouse Brain Atlas (Paxinos et al., 2007) were used to determine matching planes of section between groups (anatomical landmarks used: corpus callosum, hippocampus, and subcortical structures). Once images were selected, regions of interest (ROIs) were measured using the ImageJ (NIH) electronic micrometer function by trained researchers blind to treatment conditions, as previously reported in Abbott et al. (2016). In brief, cortical thickness was measured with respect to the cortical sheet, by drawing perpendicular lines from the most superficial region of layer I to the deepest region of layer VI. Cortical regions measured include the frontal cortex (the boundary of layer ^2/3^ of the secondary motor area to boundary of layer ^2/3^ of the orbital area), prelimbic cortex, primary somatosensory cortex (S1), primary auditory cortex (A1), and primary visual cortex (V1).

### Dendritic spine density measurements

P20 and P30 brains were hemisected and placed into a modified Golgi-Cox solution (Bayram-Weston et al., 2016; Zaqout and Kaindl, 2016) for 14 days in the dark at room temperature. Brains were then removed from the solution and placed in 30% sucrose in PBS for 2 days. Brains were then embedded in 5% agarose and sliced on a vibratome at 100 μm and mounted on subbed slides. Slides were allowed to dry for 2–3 days before developing. Slides were dipped in distilled water for 10 min, then 20% ammonia for 10 min, then distilled water for 10 min, then 70, 95, and 100% ethanol (EtOH) for 5 min each, and xylenes for 40 min. Slides were then immediately coverslipped with permount solution. Images of dendritic spines, of pyramidal cells in layer IV/V of the Prelimbic and Frontal cortices, were then imaged using a 630X oil immersion objective on a Leica Dmi8 bright field stereoscope using an attached Leica DFC 450C camera. Dendritic spine density was calculated for the entire length of the dendrites using ImageJ by an experimenter blind to condition. Counted spines were then divided by the length of the dendrite measured, then an average of dendritic spines was taken for each mouse as multiple neurons were sampled from each individual subject. In depth dendritic spine staining methodology has been previously described elsewhere (Bottom et al., 2022).

### Behavioral assays

Due to higher than zero BEC levels in LEE pups at wean, behavioral assays were only performed at P30. Therefore the 10-day post wean period was considered a “wash out” period in the paradigm. Mice were subjected to a maximum of two behavioral tests during the testing period with the forced swim test (FST) always being last due to the high-stress nature of the test. All behavioral analyses and scoring were performed and analyzed by trained researchers blind to experimental conditions. All apparatuses were cleaned using Virkon before and after each testing session.

#### Elevated plus maze

The elevated plus maze (EPM) has been historically employed to measure anxiety-like behaviors in rodents (Handley and Mithani, 1984; Rodgers and Dalvi, 1997). Notably, young CD-1 mice are known to contradict this measure of anxiety-like behaviors and they typically interact with the lower anxiety-associated metrics of this assay at higher portions; therefore, this behavior is thought to be considered risk-taking behavior (Macrì et al., 2002). This test has been used in our laboratory’s PrEE mouse model (Bottom et al., 2022). In a dimly lit room, we employed the use of a plus “+” shaped apparatus that is designed to provide test mice with two different arm environments (arm specifications; 54 cm wide and 30 cm long). The first arm type (closed arms) shields the mouse from the testing room using 15 cm high non-transparent panels that laterally enclose the mouse, with an opening on top of the apparatus. This provides the mouse with a shaded semi-enclosed space. The second arm type (open arms) exposes the mouse to the testing room through omission of the non-transparent panels. These arm types are arranged adjacently to one another on the apparatus, such that each environment is flanked by the opposing environment. Additionally, the apparatus is lifted 50 cm above the ground using stilts. In sum, mice were subjected to a single 5-min trial on the EPM where the mouse was placed in the center of the apparatus and could move freely for the entire testing period. The amount of time spent in each arm, as well as entries and total time was recorded. Video recordings were made of each testing session. A longer time spent in the open arms may indicate increased risk-taking behavior or active exploratory behavior.

#### Forced swim test

Designed to assess the effects of antidepressant drugs in the late 1970s (Porsolt et al., 1978), the FST was originally used to measure depressive-like behaviors (Lucki et al., 2001). More recently, studies have re-evaluated the interpretation of the test. Mouse performance in the water (either actively swimming/attempting to climb or floating immobile) has been viewed as a response to the stressful environment; the mice could respond with a passive coping style (immobility) or an active stress-coping style (swimming/climbing). The active stress coping has also been hypothesized to be related to hyperactivity (Commons et al., 2017; Conner et al., 2020; Armario, 2021). This technique has been used in our laboratory previously in our PrEE mice (Abbott et al., 2018; Conner et al., 2020; Bottom et al., 2022). Mice were placed in an acrylic glass cylinder (30 cm in height and 12 cm in diameter) filled to two-thirds total volume with room temperature (27°C) water for 6 min. The initial 2 min were an acclimation period and the remaining 4 min (240 s) were video-recorded and the time in which the animal was immobile in the water was recorded. Mice had light placed directly above them throughout the testing period and no more than two experimenters were allowed to be present during the testing period. Percentage of time spent immobile was calculated for each mouse.

#### Accelerated rotarod

The accelerated rotarod (AR) test was used to examine motor ability, learning, grip strength, and coordination (Rustay et al., 2003; Buitrago et al., 2004). This test has been used in our laboratory’s PrEE mouse model (Abbott et al., 2018; Bottom et al., 2022). Briefly, the mice were subjected to four, 5-min trials on the rotarod apparatus with each trial separated by a 10-min interval. The AR (Ugo Basile; Germonio, Italy) consists of a rod (diameter 28.5 mm) that rotates and gradually increases speed from 4 to 40 rpm. Mice are scored for the amount of time they are able to stay balanced on the AR. If they are able to maintain balance for the entire trial length, they are given a perfect score of 300 s.

### Statistical analyses

All statistical analyses were completed using R (v4.1.2; R Core Team, 2021). Between-subjects tests were carried out using ANOVA with Type III sums of squares (*via* the car package, v3.0.12; Fox and Weisberg, 2019). Repeated measures tests were performed using multilevel models *via* the lme4 R package (v1.1.27.1; Bates et al., 2015). Planned comparisons and simple effect tests were carried out using the emmeans R package (v1.7.2; Lenth, 2022).

## Results

### Model verification: Blood ethanol concentration and blood plasma osmolality in dams and pups

**FIGURE 2:**
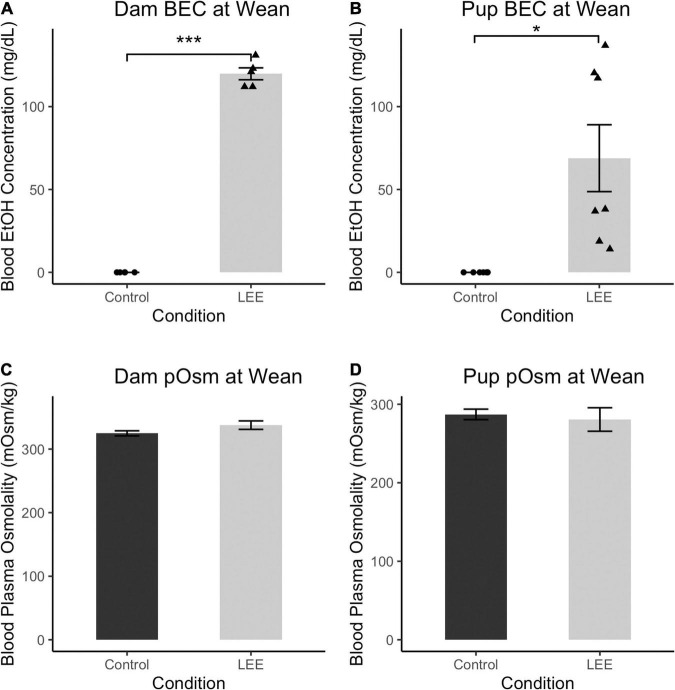
Blood ethanol concentration and pOsm measurements. **(A)** BEC measurements in Control and LEE dams at wean after a 14 day exposure to water (control) or 25% EtOH. LEE mice exposed to 25% EtOH had an average BEC of 119.8 mg/dL compared to controls which had a BEC of 0 mg/dL (*N* = 10). **(B)** BEC measurements in Control and LEE pups at wean after dams were exposed to water or 25% EtOH for 15 days. LEE pups had greater BECs (68.9 mg/dL on average) compared to controls at 0.0 mg/dL (*N* = 14). **(C)** No significant differences observed between control (*M* = 324.8 mOsm/kg, SD = 8.8 mOsm/kg) and LEE (*M* = 337.7 mOsm/kg, SD = 16.3 mOsm/kg) dam plasma osmolality (pOsm) at wean (*N* = 11). **(D)** No significant differences in pup pOsm at wean between control (*M* = 287.1 mOsm/kg, SD = 20.0 mOsm/kg) and LEE (*M* = 280.6 mOsm/kg) offspring (*N* = 16; *p < 0.05, ***p < 0.001). **(A,B)** Triangles represent individual data points taken for each experimental condition. Data expressed as mean ± SEM.

To ensure adequate maternal intake of ethanol, we measured BEC at wean. As expected, at wean, LEE dams had significantly greater BEC when compared to control dams, *t*(4) = 33.30, *p* < 0.001 (Figure 2). Additionally, to assess maternal hydration during the ethanol self-administration period, we measured dam blood plasma osmolality (pOsm). No significant differences in pOsm were found between LEE and control dams at wean, *t*(7.95) = 1.66, *p* = 0.1366, suggesting similar levels of hydration in dams across conditions. Ethanol treated dams showed lower caloric consumption and body weights when compared to control dams. From P6 through P20, LEE dams consumed fewer calories from food and ethanol combined (*M* = 75.1, SD = 9.6, 95% CI [68.3, 82.0]) than control dams (from food alone; *M* = 98.5, SD = 11.6, 95% CI [87.8, 109.2]), *t*(11.37) = 4.39, *p* = 0.001. At wean, LEE dams (*M* = 37.9 g, SD = 4.4 g, 95% CI [35.5, 40.7]) weighed less than control dams (*M* = 46.0 g, SD = 4.7 g, 95% CI [42.7, 49.2]), *t*(12.53) = 3.59, *p* = 0.003. In the case of the ethanol treated dams in the current study, they engaged in higher rates of infanticide and cannibalism [from P6 through P20, more of the LEE dams’ pups died (*M* = 5.2, SD = 3.3, 95% CI [3.0, 7.4]) than control dams’ (*M* = 1.3, SD = 1.4, 95% CI [0.2, 2.4]), *t*(14.08) = 3.49, *p* = 0.004], which would reduce their requirements to produce milk, and, to some degree, compensate for lower food intake.

Lactational ethanol exposure pups at wean, as anticipated, had greater BEC than control pups, *t*(6) = 3.41, *p* < 0.014, although considerable variation was observed between individual measures. We endeavored to account for this variation by examining the relationship between both litter size and pups’ sex on LEE pups’ BEC at wean. Neither litter size [*t*(6) = 0.34, *p* = 0.742] nor sex [*t*(6) = 0.49, *p* = 0.642], however, was a significant predictor of BEC. Nevertheless, additional possible explanations for the increased variability are discussed in the section on study limitations and future directions.

There were no significant differences in blood plasma osmolality (pOsm) found between LEE and control pups at wean, *t*(8.38) = 0.40, *p* = 0.700, also suggesting similar levels of hydration in pups across conditions. These results confirm that non-zero levels of EtOH intoxication occur in LEE dams and pups at wean. Furthermore, these results indicate no disparity in dam or pup pOsm due to the exposure paradigm.

### P20 and P30 pup gross measurements

**FIGURE 3:**
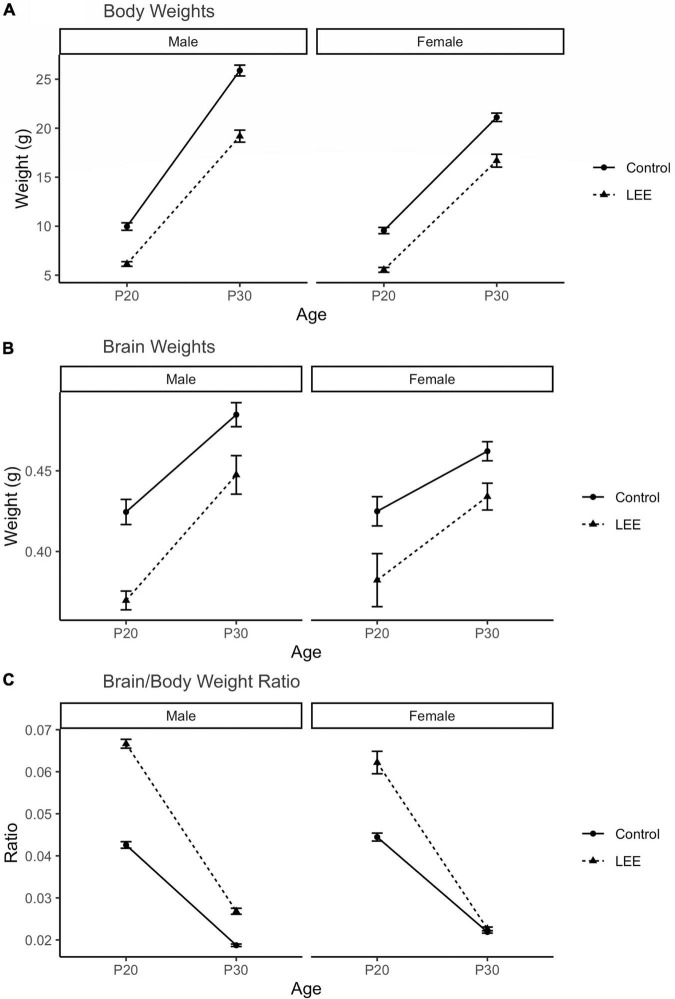
Offspring measures: Gross development. **(A)** A significant (*p* < 0.001) reduction in body weight for LEE pups was observed in every age and sex group, when compared to controls (*N* = 310). **(B)** Significant reductions in brain weights were observed for LEE males at P20 (*p* = 0.003) and P30 (*p* = 0.0332) developmental time points. However, significant reductions were only observed in P20 LEE females (*p* = 0.0085) and no significance is observed in P30 LEE females (*p* = 0.1184) compared to controls (*N* = 70). **(C)** Significant increases to the brain/body ratio are observed in LEE males at both developmental time points. Significant increases to the brain/body ratio were only observed in P20 LEE females and not P30 females as compared to controls (*N* = 70). Data expressed as mean ± SEM.

To examine the ability of our exposure paradigm to produce gross alterations in pup central nervous system (CNS), and overall development, we evaluated body and brain weights, body-brain weight ratio (Figure 3), and cortical length measurements (Figure 5) at P20/P30 and by sex.

**FIGURE 4:**
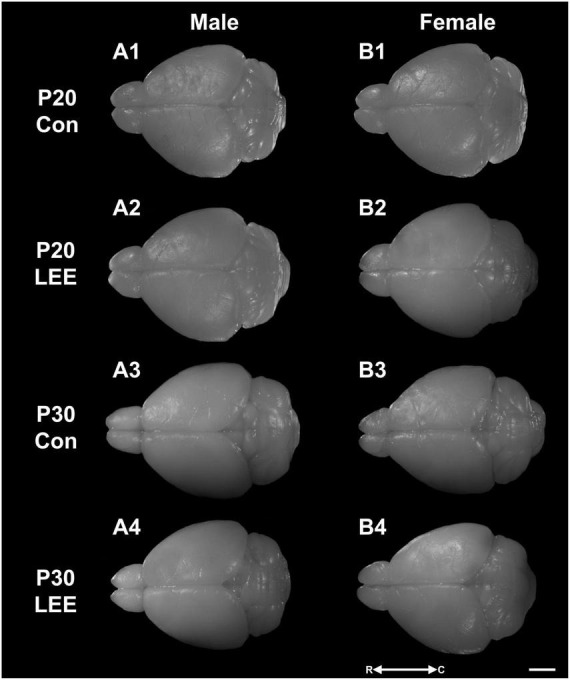
Dorsal views. Representative images of perfused and extracted brains of male **(A1–A4)** and female **(B1–B4)** pups at P20 **(A1,A2,B1,B2)** and P30 **(A3,A4,B3,B4)** after dams were exposed to water **(A1,B1,A3,B3)** or EtOH **(A2,B2,A4,B4)** for 14 days. Images oriented rostral (R) to the left and caudal (C) to the right. Scale bar, 1 cm.

**FIGURE 5:**
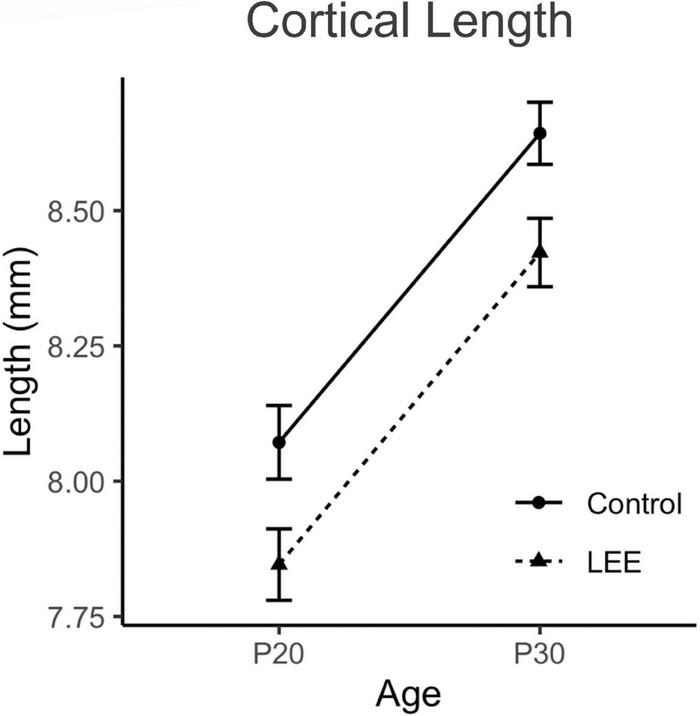
Cortical lengths. No significant differences in cortical length were observed between control and LEE pups at P20 or P30 (*N* = 70). Data expressed as mean ± SEM.

A three-way, condition (Control vs. LEE) × age (P20 vs. P30) × sex (male vs. female) ANOVA (Type III SS) identified a three-way condition × age × sex interaction on pups’ weight, *F*(1,302) = 4.22, *p* = 0.0409 (Figure 3). In light of this three-way interaction, lower order interactions and main effects should be considered with caution. Nevertheless a two-way age × sex interaction was also present, *F*(1,302) = 31.02, *p* < 0.001, as was a main effect of condition, *F*(1,302) = 58.45, *p* < 0.0001, and age *F*(1,302) = 434.23, *p* < 0.0001. To examine the three-way interaction, the two-way condition × age interactions were examined separately for male and females. For males, the two-way interaction was significant, *F*(1,302) = 12.36, *p* = 0.005, indicating that the effect of condition was greater at P30 than at P20. For females, the two-way interaction failed to reach significance, *F*(1,302) = 0.22, *p* = 0.637. Sidak corrected planned comparisons were carried out to examine the difference between the weight of the control and LEE pups at each combination of age and sex. These indicated that control pups weighed more in all four combinations: P20 male, *t*(302) = 7.343, *p* < 0.0001, P30 male, *t*(302) = 10.602, *p* < 0.0001, P20 female, *t*(302) = 7.646, *p* < 0.0001, P30 female, *t*(302) = 6.296, *p* < 0.0001.

Next, a three-way, condition (Control vs. LEE) × age (P20 vs. P30) × sex (male vs. female) ANOVA (Type III SS) identified main effects of condition, *F*(1,62) = 10.26, *p* = 0.002, and age *F*(1,62) = 9.12, *p* = 0.004 on the weight of pups’ brains. As described previously, Sidak corrected planned comparisons were carried out to examine differences between control and LEE pups weights at each combination of age and sex. Results indicated that control pups’ brains weighed more in three of the four combinations: P20 male, *t*(62) = 4.24, *p* = 0.0003, P30 male, *t*(62) = 2.72, *p* = 0.0332, and P20 female, *t*(62) = 3.20, *p* = 0.0085, but not P30 female, *t*(62) = 2.207, *p* = 0.1184.

Lastly, to consider the relationship between body and brain weight, we examined the ratio of pups’ brain to body weight *via* a three-way, condition (Control vs. LEE) × age (P20 vs. P30) × sex (male vs. female) ANOVA (Type III SS). This analysis identified main effects of condition, *F*(1,62) = 128.75, *p* < 0.001, and age *F*(1,62) = 243.95, *p* < 0.0011 on the on the ratio of pups’ brain to body weight. These main effects, however, should be considered in light of interactions between age and condition, *F*(1,62) = 61.82, *p* < 0.001, and sex and condition, *F*(1,62) = 8.47, *p* = 0.005. The age × condition interaction provided evidence that the effect of exposure to EtOH diminished between P20 (*M* = 0.022, 95% CI [0.018, 0.023]) and P30 (*M* = 0.004, 95% CI [0.002, 0.007]), and the sex × condition interaction provided evidence that the effect of exposure to EtOH was greater for male (*M* = 0.016, 95% CI [0.014, 0.019]) than female (*M* = 0.009, 95% CI [0.007, 0.012]) pups. As described previously, we carried out Sidak corrected planned comparisons to examine the difference between the ratio of pups’ brain to body weight in the control and LEE pups at each combination of age and sex. These indicated that LEE pups’ brain-body weight ratio was greater in three of the four combinations: P20 male, *t*(62) = 15.88, *p* < 0.001, P30 male, *t*(62) = 5.03, *p* < 0.001, and P20 female, *t*(62) = 11.35, *p* < 0.001, but not P30 female, *t*(62) = 0.49, *p* = 0.981.

To examine cortical length (Figures 4, 5), we performed a three-way, condition (Control vs. LEE) × age (P20 vs. P30) × sex (male vs. female) ANOVA (Type III SS) that identified main effects of condition, *F*(1,68) = 5.52, *p* = 0.022, and age *F*(1,68) = 12.69, *p* = 0.001 on the length of pups’ brains. As described previously, Sidak corrected planned comparisons were carried out to examine the difference between the weight of the control and LEE pups at each combination of age and sex. None of these comparisons indicated a significant difference between the lengths of control and LEE pups’ brains: P20 male, *t*(68) = 1.00, *p* = 0.7860, P30 male, *t*(68) = 2.27, *p* = 0.1015, P20 female, *t*(68) = 2.35, *p* = 0.0843, and P30 female, *t*(68) = 1.37, *p* = 0.5370.

Altogether, these results suggest that our exposure paradigm produces long-lasting gross alterations in CNS and general development in the LEE pups.

### P20 and P30 pup cortical neuroanatomical measurements

**FIGURE 6:**
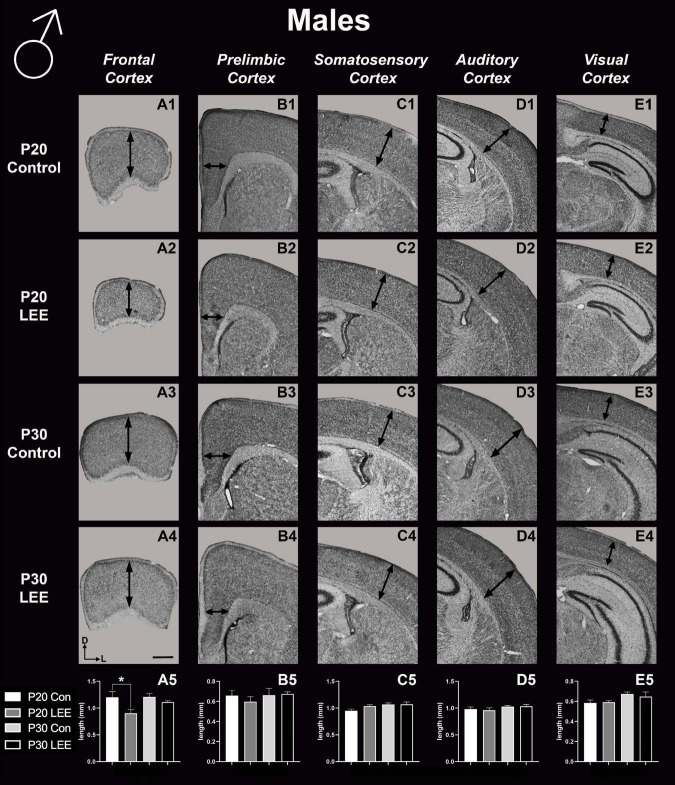
Cortical thickness measurements – males. High magnification coronal sections of Nissl-stained hemisections. Measurements include frontal cortex (**A1–A5**; *N* = 21), prelimbic cortex (**B1–B5**; *N* = 16), somatosensory cortex (**C1–C5**; *N* = 22), auditory cortex (**D1–D5**; *N* = 19), and visual cortex (**E1–E5**; *N* = 28). No significant differences between control and LEE males, except in the frontal cortex at P20 (A5, *p* = 0.0235). Data expressed as mean ± SEM. Images oriented dorsal (D) up and lateral (L) to the right. *Indicates *p* < 0.05. Scale bar, 1 mm.

**FIGURE 7:**
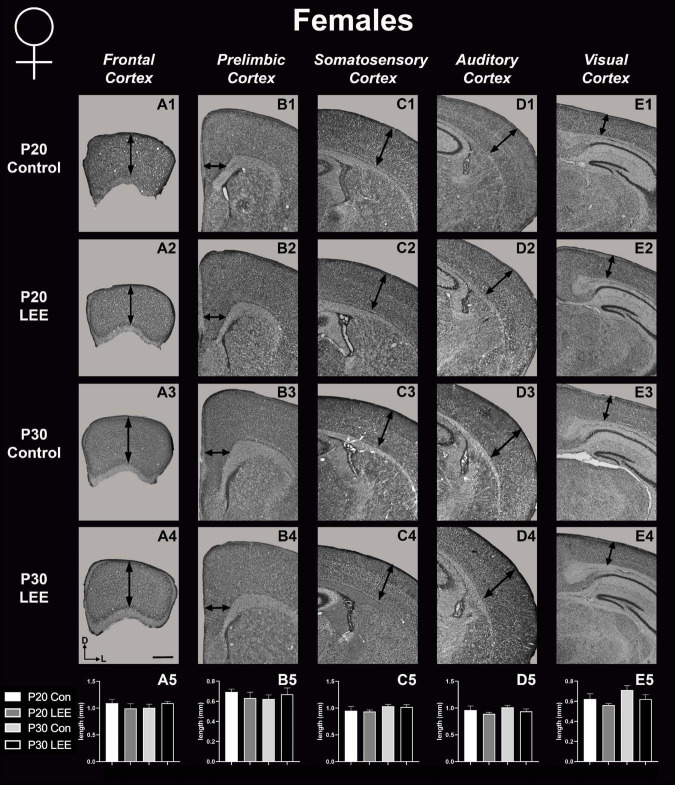
Cortical thickness measurements – females. High magnification coronal sections of Nissl-stained hemisections. Measurements include frontal cortex (**A1–A5**; *N* = 19), prelimbic cortex (**B1–B5**; *N* = 11), somatosensory cortex (**C1–C5**; *N* = 26), auditory cortex (**D1–D5**; *N* = 20), and visual cortex (**E1–E5**; *N* = 20). No significant differences between control and LEE pups. Data expressed as mean ± SEM. Images oriented dorsal (D) up and lateral (L) to the right. Scale bar, 1 mm.

To assess the effects of the exposure paradigm on cortical thickness development, we measured from five distinct regions (frontal, prelimbic, somatosensory, auditory, and visual cortices) in Nissl-stained coronal sections in both LEE and control pups at both milestone dates (Figures 6, 7). We carried out three-way condition (Control vs. LEE) × age (P20 vs. P30) × sex (male vs. female) ANOVAs (Type III SS) on the cortical thicknesses of pups’ brains in each region. In these analyses, none of the main effects or interactions were significant although the main effect of age in the visual cortex trended toward greater thickness at age P30 (*M* = 0.664 ± 0.0173) than at age P20 (*M* = 0.590 ± 0.0171), *F*(1,33) = 3.22, *p* = 0.0820. The corresponding Sidak-corrected planned comparisons we carried out to examine the difference between the cortical thickness in the control and LEE pups at each combination of age and sex also failed to show significant differences with the exception of the frontal cortex in the P20 male pups, *t*(34) = 2.94, *p* = 0.0235 (Figure 6A5).

These results suggest that there were only modest alterations to frontal cortical thickness in the development of the LEE mice.

### Dendritic spine measurements

**FIGURE 8:**
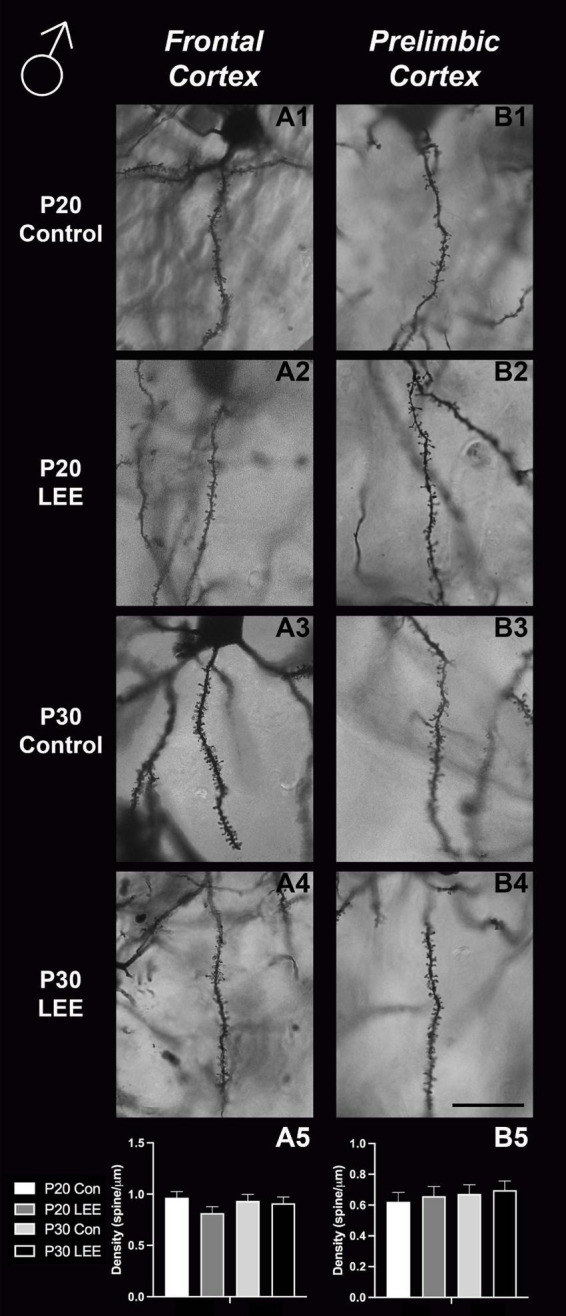
Dendritic spine density – males. Representative images of secondary dendrites of pyramidal cells in layers 4/5 of the frontal and prelimbic cortices of male control **(A1,B1,A3,B3)** and LEE **(A2,B2,A4,B4)** pups at P20 and P30. Comparison of dendritic spine density of males indicated no significant differences in frontal (**A5**; *N* = 14) and prelimbic (**B5**; *N* = 16) cortices. Data expressed as mean ± SEM. Scale bar, 250 μm.

**FIGURE 9:**
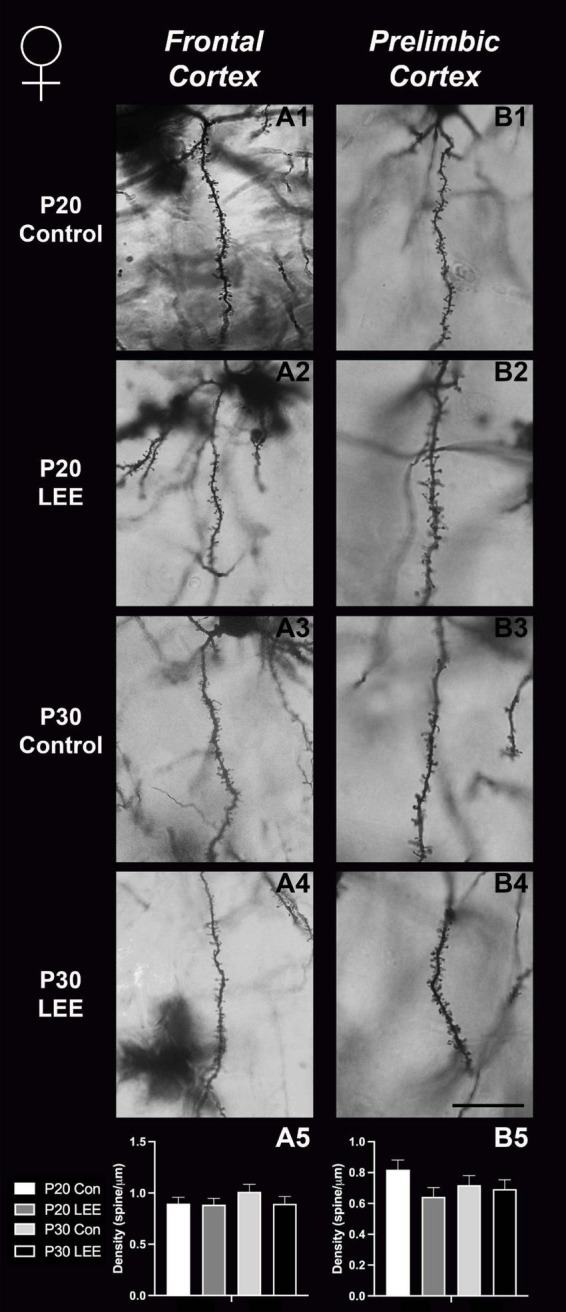
Dendritic spine density – females. Representative images of secondary dendrites of pyramidal cells in layers 4/5 of the frontal and prelimbic cortices of female control **(A1,B1,A3,B3)** and LEE **(A2,B2,A4,B4)** pups at P20 and P30. Comparison of dendritic spine density of males indicated no significant differences in frontal (**A5**; *N* = 16) and prelimbic (**B5**; *N* = 16) cortices. Data expressed as mean ± SEM. Scale bar, 250 μm.

An analysis on dendritic spine density (spines/um) was employed to explore the impact of our exposure paradigm on spine density at both milestone dates *via* Golgi-Cox-stained coronal sections (Figures 8, 9). Because we measured spinal density on multiple dendrites from individual mice, the data were analyzed using a multilevel model in which condition (Control vs. LEE) × age (P20 vs. P30) × sex (male vs. female) were fixed factors and mouse was included as a random factor. In prelimbic cortex, this analysis indicated a main effect of sex on spinal density (male, *M* = 0.662 spines/μm ± 0.0305; female, *M* = 0.719 spines/μm ± 0.0304), *t*(26.24) = 2.26, *p* = 0.0326. There was also a trend toward an effect of condition (control, *M* = 0.708 spines/μm ± 0.0307; LEE, *M* = 0.673 spines/μm ± 0.0302), *t*(23.73) = 2.05, *p* = 0.0517, and an interaction between sex and condition (male LEE – control, *M* = 0.0308 spines/μm ± 0.0610; female LEE – control, *M* = −0.1011 spines/μm ± 0.0607), *t*(24.97) = 1.73, *p* = 0.0954. Sidak-corrected planned comparisons failed to show significant differences between the spinal densities of neurons in the prelimbic cortex of control and LEE pups for either male or female pups at either age. In frontal cortex, this analysis did not indicate any significant effects or interactions, nor did any of the planned comparisons show significant differences at any combination of sex and age. Overall, these results suggest a possible modest difference between the experimental group and controls moderated by sex in prelimbic cortex, but provided no evidence of differences in dendritic spine density in frontal cortex.

### P30 behavioral analyses

To assess the impact of the exposure paradigm on behavioral development, we employed a number of behavioral tests to investigate potential differences. The included tests were: EPM, FST, and AR.

**FIGURE 10:**
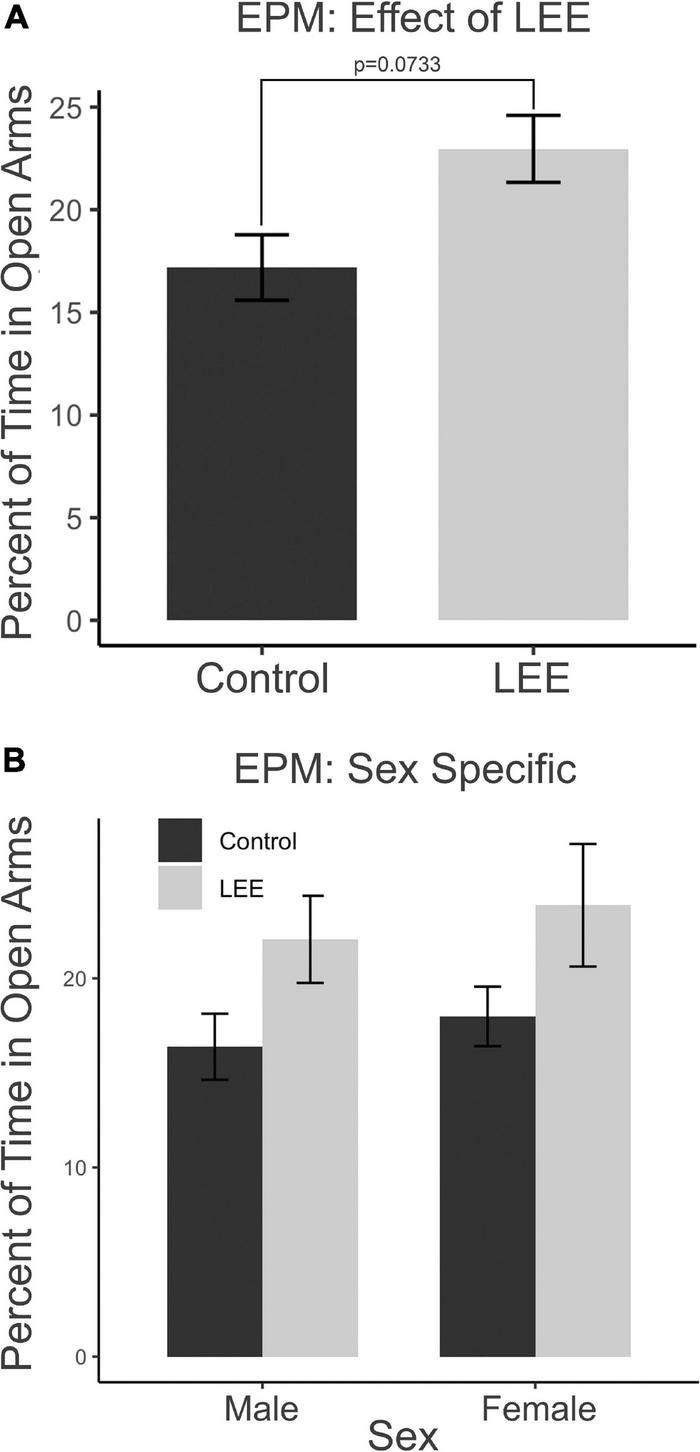
Behavioral assays at P30: EPM. **(A)** No significant differences in time spent in the open arms of the EPM when evaluated by sex and a marginal effect of condition (*p* = 0.07). **(B)** No effects observed in planned comparisons for male and female pups (*N* = 41). Data expressed as mean ± SEM.

The EPM provides a measure of anxiety-like and risk-taking behaviors. We investigated the risk-taking behaviors by recording the percent of time mice spent in the open arms of the maze (Figure 10). A two-way, condition (Control vs. LEE) × sex (male vs. female) ANOVA (Type III SS) failed to identify a significant effect of condition or sex on the time pups spent in the open arms of the maze. There was, however, a trend toward LEE pups (23.0 ± 1.63%) spending more time in open arms than control pups (17.2 ± 1.59%), *F*(1,37) = 3.40, *p* = 0.0733 (Figure 10A). Sidak corrected planned comparisons were carried out to examine the difference between the percent of time the control and LEE pups spent in open arms for male and female pups separately. These comparisons similarly failed to indicate significant differences (Figure 10B). The results suggest that LEE mice may spend more time on the uncovered arms of the apparatus compared to controls, regardless of sex (Figure 10A) suggesting the possibility of increased risk-taking behavior.

**FIGURE 11:**
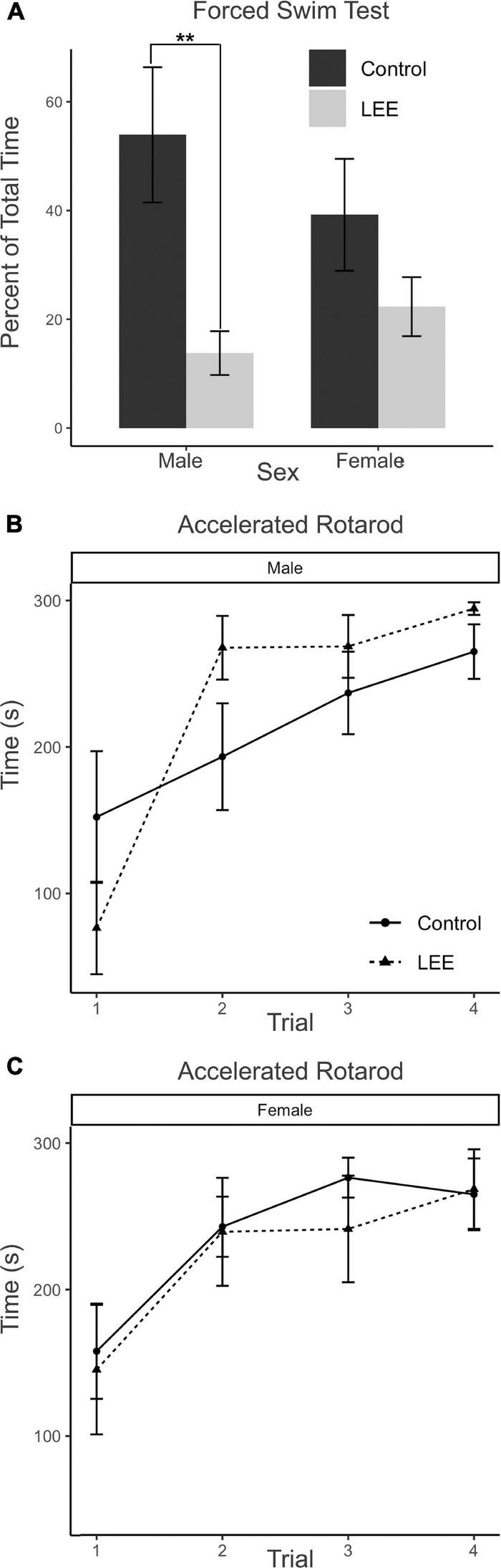
Behavioral assays at P30: FST and AR. **(A)** Male LEE pups (*p* = 0.0049) spent less time immobile than male controls in the FST. No significant differences in time spent immobile for females (*p* = 0.3588) on the FST (*N* = 34). No significant differences in performance on the AR for males **(B)** or females **(C)** (*N* = 42). Data expressed as mean ± SEM.

In the FST, immobility may be understood as a measure of passive coping behavior. A two-way, condition (Control vs. LEE) × sex (male vs. female) ANOVA (Type III SS) failed to identify a significant effect of condition or sex on the percent of time each mouse was immobile. Sidak corrected planned comparisons were carried out to examine the difference between the percent of time the control and LEE pups spent immobile for male and female pups separately. Here, it was found that male LEE pups spent less time immobile than male control pups, *t*(30) = 3.31, *p* = 0.0049 (Figure 11A). For female pups, however, the difference between the time spent immobile in the two groups was not significant, *t*(30) = 1.31, *p* = 0.3588.

The AR test measures motor ability, balance, coordination, and learning through repeated measures. Because the AR task extends across four trials for each mouse, the data were analyzed using a multilevel model in which condition (Control vs. LEE) × sex (male vs. female) × trial (1–4) were fixed factors, and mouse was included as a random factor. The analysis indicated a main effect of trial, χ^2^(3) = 104.67, *p* < 0.001. Planned polynomial contrasts showed significant linear [*t*(114) = 3.54, *p* = 0.0006] and quadratic [*t*(114) = 2.15, *p* = 0.0338] effects of trial as well as a three-way interaction between condition, sex, and the quadratic trial contrast [*t*(114) = 2.07, *p* = 0.0405]. This interaction can be understood by considering the pattern of the effect of condition across trials for males (trial 1 *M* = 75.76, 95% CI [−4.29, 155.81]; trial 2 *M* = −74.39, 95% CI [−154.44, 5.66]; trial 3 *M* = −31.77, 95% CI [−111.82, 48.28]; trial 4 *M* = −29.40, 95% CI [−109.45, 50.65]), and for females (trial 1 *M* = 12.576, 95% CI [−71.04, 96.17]; trial 2 *M* = 3.48, 95% CI [−80.13, 87.09]; trial 3 *M* = 35.05, 95% CI [−48.56, 118.66]; trial 4 *M* = −3.58, 95% CI [−87.19, 80.03]). Other main effects and interactions did not reach significance (Figures 11B, C). Additional Sidak corrected planned comparisons between adjacent trials within each combination of sex and condition yielded significant differences between trials 1 and 2 for Control, *t*(114) = 2.68, *p* = 0.0417, and LEE, *t*(114) = 2.97, *p* = 0.0191, females (Figure 11C) and for LEE males, *t*(114) = 6.61, *p* < 0.0001 (Figure 11B).

Overall, these data suggest that our exposure paradigm generates behavioral aberrations at P30 including increased risk-taking behaviors in LEE mice regardless of sex as well as abnormal stress regulation, active stress-coping styles and/or hyperactivity in male LEE mice.

## Discussion

Fifty years ago, several physicians at the University of Washington Medical School studied a small group of children who exhibited a particular set of developmental delays. The commonality among the children was that they were all born to alcoholic mothers. This was the first of many studies that aimed to identify and understand the condition that would be later known as Fetal Alcohol Syndrome (FAS) (Jones et al., 1973). Our laboratory has studied the effects of PrEE for over 10 years now and although we have gained insight on FAS, or its spectrum disorder, FASD, our work was limited to prenatal exposures. Unfortunately, maternal alcohol consumption may continue during pregnancy, or if the mother abstained from drinking while pregnant, it may begin in the early postnatal period. Many new mothers report that after 9 months of abstinence, they begin to drink again after the baby is born (Jagodzinski and Fleming, 2007). The advice by physicians for drinking alcohol while breastfeeding is quite variable, and this presents a possible health issue for infants of drinking mothers. In fact, the CDC warn against heavy drinking during breastfeeding but suggest that “moderate consumption of alcohol” is not harmful to offspring (Centers for Disease Control and Prevention, 2022b). Compared to research on prenatal alcohol exposure, studies examining the effects of maternal drinking during lactation are mostly limited to epidemiological reports with a paucity of papers in animal models where changes in the developing nervous system are investigated. Thus, we developed a novel postnatal alcohol exposure model in breastfeeding mice, using the murine strain utilized in our PrEE studies. In this LEE model, we demonstrate that maternal consumption of alcohol while breastfeeding can induce gross developmental deficits in LEE pups including decreased body weights, brain weights, and cortical lengths. Additionally, we discovered some sex-specific, LEE-related phenotypes in the neuroanatomy of the frontal lobe and prelimbic cortex, as well as behavioral deficits in stress-coping styles and risk-taking behaviors in LEE offspring. Our findings that postnatal, indirect ethanol exposure (as modeled by our lactational experimental paradigm) can negatively impact various aspects of development represents an important advancement in solidifying the significance of conscientious, informed parental care.

### A novel murine lactational ethanol exposure model: Impact of LEE on gross anatomical changes in offspring

Our results suggest that ethanol exposure *via* lactation is correlated with reduced body weights in both males and females at P20 and 30. These findings are consistent with human studies where children exposed to ethanol through contaminated breast milk can have consistently lower body weights and growth trajectories (May et al., 2016). Although there is a paucity of rodent data on offspring outcomes after ethanol exposure *via* lactation, a study from Vilaró et al. (1987) reported a reduction in body weight of ethanol-exposed rats after a period of maternal ethanol consumption while nursing her pups. In terms of brain size and morphology, we find some sex-specific effects of LEE in our model. Specifically, while LEE males show sustained low brain weights compared to controls at P20 and P30, LEE females only show deficits in brain weights at P20, with recovery to control weights by P30. Thus, LEE females show a faster rate of recovery when compared to males.

Few rodent models have examined brain weight changes in LEE mice; however, one study reported a decrease in weights of the forebrain, cerebellum, and brainstem in alcohol treated pups (Chen et al., 1998). When examining PrEE paradigms, sustained reductions in body weight and brain weights are observed from P0 to P50 in mice, consistent with findings in LEE offspring (Abbott et al., 2016; 2018). This suggests that LEE and PrEE may impact brain and body growth through similar mechanisms.

Considering the sustained growth retardation in PrEE and LEE mice, the reduction of body and brain weights might be due to the gut’s inability to efficiently extract nutrients when alcohol is ingested. Acute and chronic ethanol administration results in a reduction of protein synthesis in the small intestine (Rajendram and Preedy, 2005) and can block absorption of micro- and macronutrients (Seitz and Homann, 2001; Seitz and Suter, 2002). Additionally, nutrient deficiency has the potential to manifest in epigenetic changes, as seen in the populations affected by the Dutch Hunger Winter (Dutch Famine) (Heijmans et al., 2008). We found that, in our PrEE model, epigenetic modifications occurred *via* changes in DNA methylation, which led to epigenetic and heritable phenotypes spanning three generations of mice (Abbott et al., 2018). It is possible that examination of epigenetic markers in LEE mice could provide further insight into mechanisms underlying LEE-induced phenotypes.

### Impact of LEE on cortical length

In mammals, much of our sophisticated behavior, including language, sociability, decision making, and even fine motor skills and coordination, originates with complex functions of cells within the neocortex. In FASD or other alcohol-induced conditions, the abnormal phenotypes in humans are often related to presumed dysfunction within the neocortex (El Shawa et al., 2013). Thus, we chose to focus our study of the novel LEE model on development of the neocortex and the behaviors that are mediated, to some extent, by its function. To begin, we measured cortical length at both P20 and P30 ages in male and female LEE and control mice. We found that while the cortex expanded in length significantly from P20 to P30 in all mice, LEE cortices remained consistently smaller, regardless of sex. Few rodent models have examined the impact of LEE on cortical development, and, to our knowledge, there are no studies that specifically measure cortical length after LEE. Similarly, studies from our laboratory demonstrated a reduction in cortical length in PrEE mice (El Shawa et al., 2013; Abbott et al., 2018). As the cortex continues to grow and develop from birth to puberty in mice, we posit here that alcohol exposure *via* lactation may lead to apoptosis, increased oxidative stress, and interference with the activity of growth factors as is suggested for prenatal exposures (Goodlett and Horn, 2001).

### Neocortical thickness

In mice, neocortical lamination is present by around P5, when barrels become apparent in later IV of somatosensory cortex. According to a comprehensive set of papers from our laboratory, the areal patterning period ends around this time, P5–6, when cortical areas have adult-like connections and lamination. Beyond P6, cortical thickness continues to increase, although the changes are minimal (Dye et al., 2011a,b). Here, we measured cortical thickness across several neocortical sensory and motor regions at P20 and P30 in LEE and control mice. Given that the frontal cortex develops later than other cortical regions, and that the time of exposure is after the areal patterning period closes, it is not surprising that the only LEE-related phenotype we found was a reduction in cortical thickness in the frontal cortex of P20 LEE males. This phenotype was recovered by P30 in the LEE male mice. Subsequent measurements in prelimbic, somatosensory, auditory, and visual cortices, at both milestone dates, produced no observable differences. Few rodent models have examined the effects of LEE on neocortex, and to our knowledge there are no studies that examine cortical thickness changes after LEE. There are, however, reports of alcohol-induced changes in cortical thickness measures after PrEE. Our laboratory demonstrated changes spanning from birth to P50 in cortical thickness measures in the brains of PrEE mice (Abbott et al., 2016). PrEE models impact cortical thickness at a higher extent due to exposure during gestation, as this is the primary time when the cortex develops layer-specific organization of cell types and matures from a simply organized, single layer to a complex 6-layered structure. As the lactational exposure occurs after cortical areas subdivision and lamination, the exposure timing may be too late in development to induce significant changes in neocortical thickness.

### LEE and dendritic spine densities in frontal cortex

Through Golgi-Cox staining we aimed to evaluate the impact of LEE on dendritic spine densities, as ethanol exposure has the potential to alter synaptogenesis (Adams et al., 2022) and synaptic pruning (Kyzar and Pandey, 2015; Kyzar et al., 2016). In typically developing mice, cortex wide synaptic pruning has been reported to reach its peak 14–21 days postnatal (Lewis, 2011). In early alcohol exposure models, acute exposures led to increased dendritic pruning in the prefrontal cortex, resulting in significant synapse loss (Socodato et al., 2020). Also, acute ethanol exposure during synaptogenesis (from P5 to P7) led to drastically decreased spine densities in the caudate/putamen, however, these densities recovered to normal levels by around P30 (Clabough et al., 2022).

Here, we exposed mice to ethanol *via* lactation within this postnatal sensitive period and conducted intensive spine counts in frontal lobe ROIs in male and female mice, aged P20 and P30. While we did not find any significant changes in our measured frontal cortex spine densities, we did find a trend toward significance for prelimbic cortex (a subregion of the medial prefrontal cortex) between LEE and control mice. There were no age- or sex-dependent effects observed, but the overall reduction in spine densities observed in the prelimbic cortex of LEE mice could impact later development, and this could be possibly caused by ethanol-induced impairment to synaptogenesis or to increased synaptic pruning as the insult take places during a sensitive period for both. Of note, whether spine densities in the prelimbic cortex decrease or increase is age dependent (Galaj et al., 2020); however, alterations due to alcohol exposure have been associated with altered behavior regardless of the direction of change (Fox et al., 2020). This is not surprising given that the prelimbic cortex is a region shown to play a role in alcohol-drinking reinforcement (Engleman et al., 2020). These data are consistent with other brain areas (basal ganglia) where reductions in spine densities observed immediately after exposure seemed to reverse by 1 month of age (Clabough et al., 2022). It is possible that alterations occurred in synaptogenesis and/or pruning earlier in the exposure period and recovered by weaning when the first measures were taken.

### Impact of LEE on behavioral development

While it is important to uncover changes in the developing nervous system that are associated with ethanol exposure through lactation, understanding the potential behavioral effects of the postnatal exposure is critical. In our current study we implemented a battery of behavioral assays to examine LEE’s effect on behavioral development. The EPM is a classic way to measure anxiety in rodents (Walf and Frye, 2007). However, researchers have also looked beyond the initial interpretation of the EPM and created alternative hypotheses about how time spent in open arms versus closed arms can be interpreted. Most importantly, if an animal spends more time in the open arm, it may indicate increased risk taking or increased exploratory behavior (Macrì et al., 2002, Kozanian et al., 2018). Also, as alcohol exposure impacts fear memory learning, affecting an animal’s ability to learn a natural fear response, increased time in open arms could be from inhibited fear learning, as was observed in our PrEE model (Kozanian et al., 2018). Here, we found that, overall, LEE mice spent a significantly longer time in open arms when compared to control mice, without sex-specific effects. This suggests that exposure to ethanol *via* lactation may increase risk taking or exploratory behavior. This is consistent with exposure to ethanol *via* lactation in humans, as May et al. (2016) found that LEE children exhibited phenotypic variability consistent with FASD, with increased risk taking and cognitive deficits often present in children with FASD (Fast and Conry, 2009).

A hallmark of FASD and alcoholism is depression (Pei et al., 2011; Kuria et al., 2012) and the FST is a classic test used to detect depressive-like behaviors in animal models (Lucki et al., 2001). Like the EPM, behavioral results associated with the FST have been interpreted differently over time in the literature. Specifically, the FST test has been a successful method used to test for the effects of antidepressant drugs in that they increase the animal’s activity in the swim well (Porsolt et al., 1978). Researchers who use the test for other model systems have identified that time immobile may represent a more complex measure than simple depressive behaviors. How the animal responds to being in the swim well, with floating (immobility) or active swimming/climbing can be viewed as different adaptive reactions to the stressful environment. For example, Armario (2021) determined that mice react according to their coping style, either passively or actively, and that the FST may be a more accurate measure of coping style rather than behavioral despair. This may also be correlated with hyperactivity or possibly response to fearful stimuli. Here, we found that LEE males demonstrated reduced time immobile when compared to control males in this task, with the effect not observed in female LEE mice. This indicates that LEE may cause abnormal stress regulation and hyperactivity in males, consistent with findings in humans with FASD (Hellemans et al., 2008). For example, alcohol compromised breast milk has been found to have an activating effect in humans, as behavioral states of infants showed increased variability, such as spending less time in quiet sleep and increased crying (Schuetze et al., 2002). It is also possible that increased time spent immobile during the FST for male LEE mice could indicate alteration in fear responsivity, as we showed abnormal fear learning in our FASD model mice (Kozanian et al., 2018). This behavioral phenotype may be related to reduced frontal lobe thickness in males (Figure 6), as the frontal cortex is likely to be involved in depression (Zhang et al., 2018) and fear responsivity (Gilmartin et al., 2014).

The AR test measures motor ability, balance, coordination and learning through repeated measures. Previously, we found that rotarod performance was altered in PrEE mice; specifically, first generation PrEE mice showed deficits in performance in the first two trials compared to controls at both P20 and P30 (Abbott et al., 2018; Bottom et al., 2022). Additionally, postnatal alcohol exposure in rats can impact AR performance (Goodlett et al., 1991; Cebolla et al., 2009). In our LEE model, male LEE mice showed increased variability in performance in trials 1–2. Specifically, the change in performance was appreciably different from controls: the male LEE mice performed worse on trial 1 but showed a significantly greater degree of improvement between trials 1 and 2. After training, LEE mice performed similar to controls on the AR. In summary, male LEE mice show a greater deficit in trial 1 and showed an abrupt learning profile that differs significantly from both controls and female LEE mice.

Collectively, our results from our behavioral studies suggest LEE may impact offspring in ways similar to prenatal exposures, with increased risk-taking, hyperactivity, active stress-coping responses to environmental stressors, and transient deficits in motor coordination. Additionally, some of these LEE-induced deficits may be sex-specific.

### Critical periods, pubescence, and plasticity

Developmental critical periods are described as times when systems are “plastic” or open to change from environmental experience, such as with learning, or insult, such as with early alcohol exposure. For brain development, these are precise time points where neuronal plasticity is heightened and cortical circuits are particularly susceptible to regulation by specific sensory modalities (Jeanmonod et al., 1981). Initial explorational work in somatosensory cortical reorganization found that the removal of mouse vibrissae at birth resulted in an absence of the associated barrels (Van der Loos and Woolsey, 1973). Since then, studies have refined these events and have assigned a critical period range (first week of life in mice) for proper barrel formation (Lo et al., 2017). Additionally, the critical period for the visual system has been extensively studied. A literature review from Hooks and Chen (2007), places the critical period prior to eye opening in mice, at P0–P10. Perturbations in this period may alter cortical retinotopic maps (Hooks and Chen, 2007) along with gene expression and intra neocortical connections (Dye et al., 2012). How perturbations, insults, or changes in input impact a developing animal depends on the critical period for development in the relevant system. If events occur after closure of a critical period, the animal may be protected from detrimental harm. Unfortunately, if these events occur outside the critical period, the ability of the brain to repair itself with plasticity mechanisms may also be reduced. Understanding critical periods when comparing the impact of prenatal versus postnatal alcohol exposure, on the developing nervous system, is critical.

Compared to the effects of prenatal alcohol exposure in our mouse model of FASD, LEE has more mild phenotypes associated with the exposure, although the changes we observed in our LEE mice could have debilitating consequences if mimicked in human systems. The difference in severity of outcomes between PrEE and LEE is possibly related to critical periods for development. As described previously, much of cortical development (lamination, arealization) in the mouse reaches an adult-like state by the first postnatal week, whereas during the prenatal period and the first few days of life, the developing brain is very susceptible to change. Thus, LEE animals may be somewhat protected, when compared to PrEE, from the more severe effects of the alcohol exposure because the key elements of cortical development, particularly those regulated by gene expression, such as the development of the intricate neuronal circuitry, are near complete.

Interestingly, there are sex differences revealed in our data. Specifically, we found that LEE females recovered brain and body weights more quickly when compared to LEE males, and that frontal cortex phenotypes and atypical behavior on the FST were observed only in LEE males. Also, LEE male rotarod performance demonstrated an abrupt learning pattern that was markedly different from controls and LEE females. One hypothesis as to why LEE females fare better, when compared to LEE males, related to differences in puberty onset compared to the timing of exposure and dependent measures. Typical onset of puberty for wild-type mice begins around P28 in males, and P25 for females (Ismail et al., 2011; Molenhuis et al., 2014). Alcohol exposure prior to this period may impact the milieu of hormones that regulate onset of puberty. For example, a gradual increase of Gonadotropin Releasing Hormone (GnRH) is responsible for the typical onset of puberty; its expression is diminished in the presence of alcohol, resulting in a pubertal onset delay (Srivastava et al., 2014; Dees et al., 2017). Therefore, our model can potentially delay puberty onset in LEE mice. Considering that female mice go through puberty earlier than males, it is not surprising that LEE has a greater impact on male behavior at P30.

### Study limitation and future directions

With this study, we attempted to model offspring exposure to ethanol, naturally, *via* maternal consumption during lactation and active breastfeeding in an outbred mouse strain. With this comes limitations. For example, outbred mice have inherent variability, unlike inbred strains where genetics are controlled. However, inbred mice, such as C57BL/6 are less hardy than CD-1 mice and tend to provide inferior maternal care to their offspring. Additionally, the self-administration design of this experiment leads to variation in maternal ethanol consumption as well as milk production and composition. These factors could play influential roles in offspring outcome in addition to the impact that ethanol provides.

Another limitation is the variability in pup BEC we observed in our data. Although the variability in dam BEC was small, we believe there were several factors besides maternal ethanol levels that influenced pup BEC. The LEE pups were small at P20 and obtaining blood samples in a great enough volume for the assays was difficult. This resulted in a lower sample size. Also, by P20, some pups had begun eating chow in addition to nursing, possibly reducing ethanol intake and time from the last nursing event was variable from pups selected for analysis. Mice metabolize ethanol quickly, so increased variability in measured BEC is expected when time since the last dose is unknown. Additionally, competition for breast milk access can result in variability among pups. Also, timing of maternal alcohol consumption relative to the period of nursing that preceded the pup sampling could also introduce variability. Despite the observed variability in pup BEC, the BECs were non-zero in all LEE pups and the level was significantly higher than controls in all LEE cases.

Future studies could include shorter time periods of exposure, as human mothers sometimes breastfeed for abbreviated periods of time post-partum. Also, additional studies of gene expression analyses in the frontal cortex as well as intraneocortical connectivity would be warranted and behavior tests of fear conditioning and learning as we observed phenotypes in these domains in our PrEE models. Finally, additional behavioral assays including tests to better assess hyperactivity, such as open field and assays that can detect cognitive deficits such as Morris water maze or radial arm maze.

## Conclusion

A preponderance of evidence from researchers studying prenatal alcohol exposure and FASD led the CDC to correct its stance on drinking in pregnancy. They now clearly state “There is no known safe amount of alcohol use during pregnancy or while trying to get pregnant” (Centers for Disease Control and Prevention, 2022a). To date, the CDC has not made a similar statement regarding drinking while breastfeeding, despite research demonstrating high frequency of maternal alcohol consumption while nursing (Backstrand et al., 2004; Parackal et al., 2007; Giglia et al., 2008; Giglia, 2010; Lange et al., 2016). In their review, May et al. (2016) make a compelling argument that alcohol consumption during pregnancy can result in poor childhood outcomes.

Our data from our novel LEE model supports this notion, as our LEE model demonstrates similar phenotypes as our PrEE model; therefore, abstaining from alcohol consumption during BOTH the prenatal period and while breastfeeding is the safest option. Although the effects of LEE are mild compared to PrEE, most likely due to exposure outside critical periods for typical development, offspring exposure to ethanol *via* breast milk can have deleterious effects on developing brain and behavior and should be avoided.

## Data availability statement

The raw data supporting the conclusions of this article will be made available by the authors, without undue reservation.

## Ethics statement

This animal study was reviewed and approved by the Institutional Animal Care and Use Committee (IACUC) at the University of California, Riverside (UCR).

## Author contributions

RP assisted with research design, conducted the experiments, collected, analyzed, and interpreted the data, and wrote the manuscript. KC conducted the experiments, collected the data, and wrote the manuscript. ME contributed to the statistical analysis and interpretation of data, and wrote the manuscript. MN conducted the experiments and collected the data. KH established the research design, interpreted the data, and wrote the manuscript. All authors contributed to the article and approved the submitted version.

## Footnotes

1
brain-map.org
